# Measurement of health-related quality of life post aortic valve replacement via minimally invasive incisions

**DOI:** 10.1186/s13019-022-01964-x

**Published:** 2022-08-26

**Authors:** Mohammed Abd Al Jawad, Faisal Mourad

**Affiliations:** Department of Cardiothoracic Surgery, Ain Shams University, Abbaseya Square, Cairo, Egypt

**Keywords:** Mini AVR, Ministernotomy, Minithoracotomy, Quality of life, HRQL, RAND SF 36

## Abstract

**Background:**

Minimally invasive aortic surgery is growing in popularity among surgeons. Although many clinical reports have proven both the safety and efficacy from a surgical point of view, there are few data regarding its impact on patients’ quality of life and whether there is a difference between ministernotomy and minithoracotomy from the patient perspective.

**Methods:**

This prospective, questionnaire-based, nonrandomized study included 189 patients who underwent aortic valve replacement via a minimally invasive incision between May 2014 and December 2020 and completed at least 1 year of follow-up. The study uses the RAND SF 36-Item Health Survey 1.0 to assess and compare health-related quality of life between ministernotomy and minithoracotomy.

**Results:**

There was a statistically significant improvement in the minithoracotomy group with regard to physical functioning, role limitation due to a physical problem, and social functioning (79.69 ± 20.72, 75.28 ± 26.52, 87.91 ± 16.98) compared to the ministernotomy group (70.31 ± 22.88, 58.59 ± 31.17, 66.15 ± 27.32) with *p* values (0.0036, 0.0001, < 0.0001), respectively.

**Conclusions:**

Both minimally invasive aortic valve incisions positively impacted patient quality of life. The minithoracotomy incision showed significant improvements in physical capacity and successful patient re-engagement in daily physical and social activities. This, in turn, positively improved their general health status compared to the 1-year preoperative status.

*Trial registration*: This study was approved by the Research Ethics Committee (REC) at the Faculty of Medicine, Ain Shams University, under the number code (FWA 000017585, FAMSU R 91 /2021).

## Background

The trend toward minimally invasive surgery has extended into cardiac surgery to achieve better results for patients with the same quality as conventional median sternotomy. Aortic valve surgery has advanced significantly due to the widespread adoption of more minor invasive procedures and new technologies. Indeed, an increasing number of surgeons are treating aortic valve problems with smaller chest incisions to lessen the invasiveness of the surgical procedure while also improving clinical and cosmetic outcomes [1].

Traditionally, postcardiac surgery results were measured with classic clinical outcomes such as event freedom and overall survival. From the physician perspective, these outcomes are critical, but they may have disregarded other patient-related outcomes. As a result, the term quality of life was coined by patients [2].

Quality of life has been applied explicitly to those life concerns that are most influenced by health or illness in matters linked to health care, hence the term health-related quality of life (HRQL) [3].

HRQL evolved from the broader notion of overall quality of life and is, by definition, more focused on the components of life quality that are influenced or can be influenced directly by one's health state. Side effects of disease and treatment, treatment satisfaction, physical functioning and wellness, social functioning and life satisfaction, and mental health, including emotional wellbeing and cognitive functioning, are all examples of these factors [3].

The 36-item Short Form (36) Health Survey is a patient-reported health survey with 36 questions. The SF-36 has been thoroughly validated and comprises eight scaled scores that are the weighted sums of the items in each component. Each scale is converted to a 0–100 scale, assuming that each question is equally weighted. The lower the score is, the greater the degree of disability. The higher the score is, the less severe the disability; for example, a score of zero corresponds to a maximum disability, while a score of 100 corresponds to no disability. The eight sections are vitality, physical function, body discomfort, general health perceptions, physical role function, emotional role function, social role function, and mental health [4, 5].

This study aims to use the SF 36 questionnaire to compare the HRQL between two approaches for minimally invasive aortic valve replacement in patients.

## Methods

This prospective, questionnaire-based, nonrandomized study included all patients who underwent aortic valve replacement via a minimally invasive incision between May 2014 and December 2020.

Of the 260 previously reported patients [6], 189 were interviewed and completed the official validated Arabic version of the SF-36/RAND-36 questionnaire [7] Postoperative questionnaires were collected from those who completed at least 1 year of follow-up.

Retrospective data were collected from the registry and reanalyzed for the 189 patients who completed the questionnaire. These data included preoperative demographics, operative details, and the immediate postoperative course.

The ministernotomy group underwent a J-sternotomy from 1 cm above the angle of Luis to the 4th intercostal space. Cardiopulmonary bypass was achieved by means of central aorto-atrial cannulation. The minithoracotomy group underwent a right anterior thoracotomy via the 2nd or 3rd intercostal space. Cardiopulmonary bypass was achieved by peripheral femoral cannulation.

All surgical procedures were performed under moderate hypothermia 28–32 °C with antegrade cardioplegia.

This study was approved by the Research Ethics Committee (REC) at the Faculty of Medicine, Ain Shams University, under the number code (FWA 000017585, FAMSU R 91 /2021).

### Statistical analysis

The scoring of the questionnaire followed the RAND 36-Item Health Survey 1.0. Precoded responses were given numeric values; the higher the value was, the more favorable the outcome. Numeric values range between 0 and 100, representing a percentage of the total possible score. Then, numeric values of the same scale are averaged together to create the final eight scale scores [8] Data are reported as the mean ± SD, and *P* values > 0.05, ≤ 0.05, and < 0.0001 were considered indicative of statistical nonsignificance, statistical significance, and high statistical significance, respectively.

## Results

Both groups were comparable in terms of demographics, comorbidities, and preoperative echocardiographic data with no statistically significant differences between the groups (Table 1).

**Table 1:** Patient demographics and characteristics

Variable	Ministernotomy (n = 96)	Minithoracotomy (n = 93)	X^2^/t	*P* value
Age (mean ± SD)	58.15 ± 5.37	59.5 ± 6.7	1.531	0.128
Sex				
Female	61 (63.5%)	55 (59.1%)	0.386	0.534
Male	35 (36.5%)	38 (40.9%)		
BMI	28.27 ± 4.2	27.68 ± 5.08	0.871	0.385
Diabetes mellitus	54 (45.8%)	40 (43%)	3.312	0.069
Hypertension	52 (54.2%)	53 (57%)	0.152	0.696
NYHA class				
I–II	33 (34.4%)	46 (49.5%)	4.420	0.036*
III–IV	63 (65.6%)	47 (50.5%)		
Preoperative LVEF	61.45 ± 5.12	60.2 ± 5.34	1.643	0.102
Preoperative LV ESD (cm)	3.75 ± 0.9	3.64 ± 0.83	0.873	0.384
Preoperative LV EDD (cm)	5.71 ± 0.74	5.6 ± 0.95	0.890	0.375
Preoperative PAP (mmHg)	14.5 ± 1.95	13.95 ± 2.03	1.900	0.060
Valve affection				
Aortic stenosis	39 (40.6%)	46 (49.5%)	1.491	0.222
Aortic regurgitation	41 (42.7%)	30 (32.3%)	2.200	0.138
Combined	16 (16.7%)	17 (18.2%)	0.085	0.770

Values are shown as the mean ± SD and percentage, *SD* standard deviation, *BMI* body mass index, *NYHA* New York Heart Association, *LVEF* left ventricular ejection fraction, *PAP* pulmonary artery pressure

* statistically significant

The ministernotomy group had more ill patients with NYHA classes III–IV (63 patients vs. 47 patients, *p* value 0.036), which was statistically significant (Table 1).

All 189 patients received mechanical valves as per the patients’ request and/or surgical preference. Other operative data, including total bypass and cross clamp times, are shown in Table 2, with significantly shorter times for the ministernotomy group (Table 2).

**Table 2:** Surgical details

Variable	Ministernotomy (n = 96)	Minithoracotomy (n = 93)	X^2^/t	*P* value
Time to cannulation (min)	43.27 ± 1.68	24.7 ± 5.9	29.626	< 0.001*
Cross clamp time (min)	61.29 ± 14.33	67.3 ± 16.35	2.690	0.008*
Total bypass time (min)	93.7 ± 22.16	114.8 ± 24.07	6.273	< 0.001*
Length of incision (cm)	8.11 ± 0.87	5.71 ± 0.56	22.472	< 0.001*
DC requirement	27 (28.1%)	36 (38.7%)	2.382	0.123
Inotrope support	57 (59.4%)	70 (75.3%)	5.413	0.020*

Values are shown as the mean ± SD and percentage, *SD* standard deviation, *DC* cardioversion, *TEE* transesophageal echocardiography

* statistically significant

The postoperative course data are shown in Table 3

**Table 3:** Post-operative course

Variable	Ministernotomy (n = 96)	Minithoracotomy (n = 93)	X^2^/t	*P* value
Total ventilation hours	17.26 ± 7.3	11.57 ± 4.75	6.330	< 0.001*
Total blood loss (ml)	263.5 ± 57.24	246.21 ± 51.26	2.185	0.030*
Total blood transfusion units	1.66 ± 0.75	1.64 ± 0.69	0.191	0.850
Total ICU stay (days)	2.54 ± 1.66	2.62 ± 1.80	0.318	0.751
Re-exploration of bleeding	5 (5.2%)	8 (8.6%)	0.849	0.357
Postoperative pain, ICU (range 1–10)	4.57 ± 1.19	5.16 ± 1.06	3.595	< 0.001*
Postoperative pain, discharge (range 1–10)	1.55 ± 0.71	1.76 ± 0.58	2.223	0.027*
Postoperative pain, 30 day (range 1–10)	1.32 ± 0.59	1.51 ± 0.4	2.583	0.011*
Incidence of new atrial arrhythmia	8 (8.3%)	20 (21.5%)	6.494	0.011*
Incidence of new ventricular arrhythmia	3 (3.1%)	4 (4.3%)	0.183	0.669
Incidence of heart block requiring PPM	2 (2.08%)	5.4%)5	1.436	0.231
Postoperative cerebrovascular stroke	5 (5.21%)	4 (4.3%)	0.086	0.770
Postoperative renal impairment (requiring dialysis)	2 (2.08%)	1 (1.1%)	0.307	0.579
Superficial wound infection	3 (3.13%)	3 (3.2%)	0.002	0.968
Groin complications	0 (0.0%)	2 (2.2%)	2.087	0.149
Postoperative LVEF	54.14 ± 9.8	55.52 ± 11.4	0.893	0.373
Postoperative LV ESD (cm)	4.38 ± 2.5	4.71 ± 2.35	0.934	0.351
Postoperative LV EDD (cm)	5.29 ± 0.77	5.67 ± 0.91	3.103	0.002*
Postoperative PAP (mmHg)	17.25 ± 4.21	14.76 ± 4.52	3.920	< 0.001*
Follow up pericardial effusion requiring drainage	17 (17.71%)	13 (13.98%)	0.492	0.483
Follow up pleural effusion requiring drainage	7 (7.29%)	13 (13.98%)	2.232	0.135
Transvalvular mean pressure gradient (mmHg)	16.6 ± 3.10	19.1 ± 4.79	4.273	< 0.001*
Hospital stay	11.2 ± 2.9	10.7 ± 2.21	1.330	0.185

Values are shown as the mean ± SD and percentage, *SD * standard deviation, *PPM* permanent pacemaker

* statistically significant

The questionnaire was completed by 189 patients, with 96 undergoing ministernotomy and the remaining 93 undergoing minithoracotomy.

Even though there was no statistically significant difference in how patients reported their general health status (*P* = 0.0532), the minithoracotomy group reported a statistically significant improvement in their health condition compared to a year prior (93.28 ± 11.74, 83.6 ± 22.6, *P* = 0.0003) (Table 4).

**Table 4:** SF 36 scales and health changes in ministernotomy compared to minithoracotomy

	Ministernotomy n = 96	Minithoracotomy n = 93	T test	*P* value
Physical functioning	70.31 ± 22.88	79.69 ± 20.72	2.951	*P* = 0.0036
Role limitation due to physical problems	58.59 ± 31.17	75.28 ± 26.52	3.959	*P* = 0.0001
Role limitation due to emotional problems	78.82 ± 26.1	82.35 ± 24.2	0.963	*P* = 0.3366
Energy/Fatigue	66.93 ± 19.95	66.02 ± 10.49	− 0.391	*P* = 0.6965
Emotional well being	78.27 ± 17.19	74.76 ± 17.15	− 1.405	*P* = 0.1617
Social functioning	66.15 ± 27.32	87.91 ± 16.98	6.552	P < 0.0001
Pain	78.1 ± 21.41	83.46 ± 17.42	1.884	*P* = 0.0611
General health	66.31 ± 22.22	61.02 ± 14.15	− 1.945	*P* = 0.0532
Health change	83.6 ± 22.6	93.28 ± 11.74	3.678	*P* = 0.0003

Data are reported as the mean ± SD, *P* value > 0.05 is nonsignificant, *P* value ≤ 0.05 is significant, *P* value < 0.0001 is highly significant

Physical functioning was significantly better for minithoracotomy than for ministernotomy (79.69 ± 20.72, 70.31 ± 22.88, *P* = 0.0036). In addition, the minithoracotomy group outperformed the ministernotomy group in regard to role limitation due to physical problems (75.28 ± 26.52, 58.59 ± 31.17, *P* = 0.0001) (Table 4).

In the minithoracotomy group, this was accompanied by a highly significant improvement in social functioning (87.91 ± 16.98, 66.15 ± 27.32, *P* 0.0001) (Table 4).

Other reported scales are shown in Table 4.

## Discussion

Many studies have reported clinical outcomes of both minimally invasive approaches for aortic valve replacement with inconclusive and insufficient data to tip the balance in favor of one approach over the other [9].

From a surgeon’s perspective, ministernotomy patients had shorter clamp and total bypass times, and the procedure was less technically demanding with a shorter learning curve [10].

Ministernotomy is also associated with fewer pleural and pulmonary complications than minithoracotomy, with reduced pain perception. However, this is true only for the first 30 days post-hospital discharge, where recent data show pain scales to be the same after that time [11].

For several years, minimally invasive cardiac surgery outcomes have been reported as “hard” outcomes, such as mortality, complications, total bypass and cross-clamp times, and other technical aspects. Although these are very important from a clinician point of view, this disregards the patients’ perceptions of their life postsurgery, their physical capacity to perform daily activities, their ability to re-engage in sports they like, their emotional and psychological well-being and their overall social life [12].

Sicouri and colleagues compared the preoperative and postoperative quality of life for 24 consecutive patients who underwent minimally invasive aortic valve intervention at 1 and 3 months of follow-up. Similar to our report, they proved significant increases in their cohort’s functional physical capacity, energy, and general health after 3 months compared to the preoperative baseline [13].

Sternal stability is one of the important determinants in regaining postoperative physical activity. Unfortunately, many reports comparing ministernotomy to conventional sternotomy failed to show a significant difference in stability between the two “sternotomy” approaches [14].

Sternal integrity is crucial for faster respiratory recovery, early hospital discharge and physical functioning [15].

The physical functioning scale includes daily activity, including the ability to engage in vigorous activities such as running and weightlifting, to perform moderate activities or to carry groceries, to climb several flights of stairs, to walk several blocks, to bend, and to kneel, all of which are related to sternal stability. It was no surprise in our study that minithoracotomy patients outperformed ministernotomy patients, especially regarding weightlifting, bending and kneeling.

Compared to our previous report, where ministernotomy significantly outperformed minithoracotomy regarding postoperative pain, it seems that after 1 year, patients with minithoracotomy showed greater improvement than ministernotomy, yet the difference did not reach statistical significance ([6], Table 4). This is consistent with other reports of “no difference” in pain perception after 30 days and 1 year [11].

Emotional wellbeing includes postoperative anxiety, depression, and emotional stability. These have an impact on role limitations following cardiac surgery. Although the SF 36 has three scales that assess the “emotional wellbeing,” “the role limitation due to emotional problems,” and “social functioning,” it has no specific anxiety assessment tool like the Hospital Anxiety and Depression Scale (HADS).” However, both the work of Sicouri and colleagues and our work showed improvements in the anxiety scale score following minimally invasive aortic incisions; this was associated with better improvement in the minithoracotomy group [13]. Improvements in well-being mainly contributed to how the patients perceived their wounds; smaller hidden incisions with full sternal integrity were considered more aesthetically pleasing by the patients, especially female patients [16].

The safety and efficacy of minimally invasive aortic techniques have been proven, although there is insufficient data for a preference of one incision over the other, especially in terms of postoperative patient quality of life. More extensive studies are needed to conclude such a debate. Additionally, one must consider the quality of life post-TAVI as a highly competitive approach [17].

This study has the important limitation of lacking a preoperative questionnaire assessment to measure improvement from the preoperative period to the postoperative period, lacking randomization, being a single-center report, and lacking of a full sternotomy group as a control.

## Conclusions

Minimally invasive aortic valve replacement is associated with improving patient quality of life. In addition, minithoracotomy is more closely associated with improved physical and social well-being and improved perception of general health and health change.

## Data Availability

The datasets used and/or analyzed during the current study are available from the corresponding author on reasonable request.

## Abbreviations

AVR: Aortic valve replacement
HRQL: Health-related quality of life
SF 36: Short Form 36
HADS: Hospital Anxiety and Depression Scale
TAVI: Transcatheter aortic valve implantation
